# Diagnostic tests for medullary thyroid carcinoma: an umbrella review

**DOI:** 10.1007/s12020-023-03326-6

**Published:** 2023-03-06

**Authors:** Pierpaolo Trimboli, Caterina Mian, Arnoldo Piccardo, Giorgio Treglia

**Affiliations:** 1 Clinic of Endocrinology and Diabetology, Lugano and Mendrisio Regional Hospital, Ente Ospedaliero Cantonale, 6500 Bellinzona, Switzerland; 2 Faculty of Biomedical Sciences, Università della Svizzera Italiana, 6900 Lugano, Switzerland; 3 Operative Unit of Endocrinology, Department of Medicine-DIMED, University of Padova, Padova, Italy; 4 Nuclear Medicine Department, E.O. “Ospedali Galliera”, 16128 Genoa, Italy; 5 Faculty of Biology and Medicine, University of Lausanne, 1011 Lausanne, Switzerland; 6 Division of Medical Education and Research, Ente Ospedaliero Cantonale, 6500 Bellinzona, Switzerland

**Keywords:** Medullary thyroid carcinoma, Calcitonin, Ultrasound, FNA, PET

## Abstract

**Purpose:**

To summarize the more robust evidence about the performance of tools useful for diagnosis of medullary thyroid carcinoma (MTC) such as calcitonin (Ctn) and other circulating markers, ultrasound (US), fine-needle aspiration (FNA), and other imaging procedures.

**Methods:**

This systematic review of systematic reviews was carried out according to a predefined protocol. A search string was created. An electronical comprehensive search of literature was performed on December 2022. Quality assessment of eligible systematic reviews was performed and main findings were described.

**Results:**

Twenty-three systematic reviews were included and several findings were achieved. Ctn is the most reliable diagnostic marker of MTC with no evidence of improvement with stimulation test. CEA doubling time is more reliable than Ctn in identifying MTC with poorer prognosis. US sensitivity is suboptimal in MTC and only just over half of cases are at high risk according to Thyroid Imaging And Reporting Data Systems. Cytology can correctly detect MTC in just over half of cases and measuring Ctn in washout fluid from FNA is necessary. PET/CT is useful for detecting recurrent MTC.

**Conclusions:**

Future guidelines of both thyroid nodule management and MTC diagnosis should consider these evidence-based data.

## Introduction

The prognosis of medullary thyroid carcinoma (MTC) depends strongly on the stage on diagnosis and initial treatment [1]. Since MTC derives from C cells that produce calcitonin (Ctn), the latter represents the most reliable test to diagnose MTC. However, while MTC diagnosis should be theoretically simple, it remains challenging due to several reasons. In fact, MTC is a rare tumor, a fixed Ctn diagnostic cut-off is not available, and, as a consequence, routinely testing for Ctn in patients with nodular goiter is not universally accepted. Then, the recommendations from international societies about Ctn testing are quite heterogeneous and mainly based on low-to-moderate evidence-based data [2–5]. In addition, it is worth to be mentioned that how MTC presents at ultrasound (US) is still unclear, the performance of cytological examination after fine-needle aspiration (FNA) is suboptimal, and the use of other imaging procedures (i.e., computed tomography, CT; magnetic resonance, MR; positron emission tomography, PET) remains heterogeneous depending on several factors including their availability [6]. From the clinical standpoint, despite the recommendations from international societies, it is needed to summarize the evidence-based data available in literature to furnish clinicians with complete information about the optimal management of tools to diagnose MTC.

The present umbrella review was conceived to summarize the most robust evidences about the performance of (1) Ctn and other circulating markers, (2) US, (3) FNA, and (4) imaging procedures, in diagnosing MTC.

## Methods

This umbrella review (or systematic review of systematic reviews) was carried out according to a predefined protocol. Based on the above-listed objectives, a search string was created using a combination of keywords and Boolean operators. The complete search algorithm used for the literature search was the following: (A) “medullary” OR “MTC” AND (B) “thyroid” AND (C) “systematic review” OR “meta-analysis” OR “evidence-based”. An electronical comprehensive search of literature using the above-listed search string on two bibliographic databases (PubMed/MEDLINE and Cochrane Library) was performed. The last update of literature search was 31st December 2022. No language restrictions or time limits were used. About the inclusion criteria, only systematic reviews with or without meta-analyses investigating circulating markers, US, FNA, and imaging procedures, in diagnosing MTC were eligible for inclusion. At least two reviewers independently performed the literature search, the selection of eligible systematic reviews applying the inclusion criteria mentioned above, and the data extraction. Quality assessment of eligible systematic reviews was performed according to AMSTAR-2 (A MeaSurement Tool to Assess systematic Reviews, version 2) tool. For each selected systematic review (with or without meta-analysis), information was collected about authors, year of publication, number of original articles, MTC patients included, and main findings.

## Results

### Articles retrieved

According to the above algorithm of search, 159 records were retrieved and 23 systematic reviews published from 2010 to 2022 were selected according to the predefined inclusion and exclusion criteria [7–29]. Main findings of eligible evidence-based articles are summarized here below. The quality assessment of eligible systematic reviews is available in the Supplementary material.

## Markers

### Calcitonin

Two systematic reviews have been published on the accuracy of measuring Ctn to diagnose MTC in patients with thyroid nodules [7, 8]. The first one [7] included 16 studies and 72,368 patients with 187 MTCs, and the prevalence of disease was 0.23%. Using a cut-off for basal Ctn of 10 pg/mL, the pooled sensitivity and specificity were 100% (95% CI: 99.7–100) and 97.2% (95% CI: 95.9–98.6), respectively, and, more interestingly, the positive predictive value was 7.7%. When using 100 pg/ml as threshold, the Ctn sensitivities lowered to 42-to-100% while specificities remained between 95 and 100%. In the subgroup of studies reporting both basal and stimulated Ctn, sensitivities ranged from 82% to 100% while specificities from 99% to 100%. These results were obtained using different thresholds for stimulated Ctn (i.e., 100 pg/mL, 60 pg/mL or 50 pg/mL for females, 80 pg/mL for males). The second evidence-based report [8] encompassed 17 studies and 74’407 patients, with 203 MTCs and a disease prevalence of about 0.11%. Using basal Ctn thresholds between 4.6 and 100 pg/mL, the pooled sensitivity and specificity were 99% (95% CI: 81–100) and 99% (95% CI: 97–99), respectively. Also, pooled positive and negative likelihood ratio were 72.4% (95% CI: 32.3–162.1), and 0.01% (95% CI: 0.00–0.23), respectively. Interestingly, the meta-regression analysis identified the covariate “threshold of basal Ctn” (i.e., Ctn ≥ 10 pg/mL versus other thresholds) as an independent influencing factor while not the covariate “performing a stimulation test”.

Only one systematic review in the literature examined rationale, technical issues, and side effects of Ctn stimulation tests used to diagnose MTC [9]. It included 25 studies, 12 reporting calcium (Ca) and pentagastrin (Pg) stimulation separately in the same group and other 4 reporting Ca and Pg in combination. Side-effects were classified according to FDA 21 312.32 Code. As results, Ca stimulation was generally associated to mild side-effects, such as feeling of warmth, nausea, an altered gustatory sensation, and headache, while after Pg stimulation the side-effects were generally moderate including neck/chest tightness, nausea, and abdominal discomfort. Overall, there was only one case of life-threatening adverse event (LTAE), i.e., asystole, after Ca stimulation. When Ca and Pg stimulation were compared, the side-effects of Pg infusion were more severe and almost all patients said they preferred Ca test over Pg test. There was a gender-specific difference in side-effects with females having fewer side effects by Pg than Ca whereas males tolerated Ca better than Pg.

Other two systematic reviews were conceived to explore the matter of MTC without Ctn secretion (i.e., non-secretory MTC) [10, 11]. The first one was updated on 2014 and concerned 11 original reports describing clinical and pathological behavior of histologically-proven MTC in 18 patients (11 females, 5 males, gender not reported in 2 cases; mean age 50 yrs, range 16–73 yrs; mean tumor size 26 mm, range 0.5–80 mm). Although different Ctn assays were used for different patients, and for the same patients at different time, and different upper limits of normal were considered, Ctn was below 10 pg/mL in all but one case (i.e., 11 pg/mL). Analytical interferences, such as the “hook effect” or the presence of heterophilic antibodies, were ruled out by serum dilutions in one patient. Alternative serum markers of MTC were also considered in some patients: carcinoembryonic antigen (CEA) was above the upper reference limit in 1 of 12 cases; chromogranin A (CgA) was normal in 3 of 3 cases; and procalcitonin (PCtn) was moderately increased in the one case in which this marker was analyzed. A cytological assessment conducted in 7 of 18 patients found only 2 certain or suspected cases of MTC, while immunocytochemical findings were always positive for Ctn protein expression in the 5 patients tested. Immunohistochemistry (IHC) confirmed a certain degree of Ctn staining (from diffuse to focal) in 13/13 cases examined. Interestingly, cases of Ctn-negative MTC exhibited a heterogeneous histological and clinical landscape, like those of secretory MTC. Indeed, at histology, they varied from well differentiated (10/18) to poorly differentiated (6/18). Two of the 18 patients had micro-MTC. The clinical prognosis was mixed, with some patients achieving a long-term survival, while others (all cases of poorly differentiated MTC) experienced a rapid disease progression and died within a few years after diagnosis. The second systematic review on non-secretory MTC [11] was updated to February 2018 and included 19 reports on 49 patients (24 females, 24 males, gender not stated in 1 case; mean age 51.7 yrs, range 16–82 years; mean tumor size 63 mm, range 10–80 mm). The Ctn levels were measured at diagnosis in only 20 cases with mean value of 8.66 pg/ml (range 0.8–38 pg/mL). As for the patients’ clinical presentation (when reported) 11 patients presented with a palpable mass, 3 with neck pain, 2 with an ultrasound incidentaloma, and 8 with symptoms attributed to cancer local spread. This review [11] confirmed the previous one [10]: (i) only 56% (12/23) of cytological specimens suggested MTC; (ii) definitive pathology detected 18 cases of well differentiated MTC, and 8 cases of poorly differentiated MTC, while the cancer grade was not stated in the remaining cases; (iii) on IHC, 55% (21/38) showed diffuse or focal positivity for Ctn, while 95% (41/43) were positive for CgA; (iv) follow-up identified 11 patients as cured, while 7 had a recurrence (involving multiple organs in 2), and 9 died of their disease. Table 1 summarizes the findings of these systematic reviews.

**Table 1:** Summary of findings of calcitonin performance in diagnosing MTC and/or detecting its relapse

First author	Year	Studies included	MTC included	With/without meta-analysis	Main findings
Verbeek [7]	2020	16	187	With	Basal and stimulated Ctn measurements have high sensitivity and specificity for MTC diagnosis. However, there is insufficient evidence for routine Ctn testing in all patients with thyroid nodes, being the prevalence of disease extremely low.
Verdali [8]	2021	17	203	With	Basal and stimulated Ctn measurements are accurate for MTC diagnosis. However, the Ctn cut-off values should be considered and a wait-and-see strategy should be applied to avoid overtreatment.
Baetu [9]	2021	25	2251	Without	Ca stimulated Ctn should be considered, when appropriate. Clinical, instrumental and biochemical assessments before the test, as well as cardiac monitoring during and after the test, should be performed to avoid serious events.
Trimboli [10]	2015	11	18	Without	The diagnosis of non-secretory MTC remains a challenge. In such a context, additional markers at diagnosis together different imaging procedures during follow-up have to be used.
Gambarella [11]	2019	19	49	Without	Non-secretory MTC is an extremely rare entity. Reliable option for clinical management together with novel biomarkers for diagnosis and relapse are needed.

*MTC* medullary thyroid carcinoma, *Ctn* calcitonin, *Ca* calcium

### Other circulating markers

Six systematic reviews have reported findings of alternative markers to Ctn in diagnosing MTC and/or detecting its recurrence [12–17]. Overall, these studies summarized, with or without meta-analysis, published data about CEA, PCtn, carbohydrate antigen 19–9 (Ca 19-9), CgA, and pro-gastrin releasing peptide (Pro-GRP).

Three studies were about the detection of recurrent MTC. One systematic review [12] included 10 studies and performed a meta-analysis using 73 cases to explore the performance of both Ctn and CEA doubling time. Post-operative Ctn above upper normal reference was associated with death with Hazard Ratio 5.08 (95% CI: 0.59–43.38) and with relapse with Hazard Ratio 5.89 (95% CI: 1.70–20.45). Postoperative CEA above normal reference was also associated with death with Hazard Ratio 40.90 (95% CI: 0-∞). Postoperative Ctn and CEA doubling times were also evaluated and while Ctn with doubling time <1 year showed Hazard Ratio of 21.52 for death and 5.33 for recurrence, CEA doubling time <1 year showed Hazard Ratio of infinite for death and 6.80 for recurrence. Another study [15] was focused on PCtn performance in detecting MTC recurrence. There were 296 MTCs of which 140 with proven recurrence. The pooled sensitivity of PCtn in detecting recurrence was 96% (95% CI: 92–99%). Also, the pooled specificity was 96% (95% CI: 87–100%). The third one [17] found 14 studies about CEA, 11 about PCtn, 5 about ProGRP, 3 about Ca 19-9, and 6 about CgA, but no meta-analysis was performed. Overall, the authors concluded that Ctn is the most accurate marker, PCtn has potential to replace Ctn, and CEA can detect cancer progression.

The remaining three systematic reviews were focused on both initial diagnosis and relapse detection of MTC. One paper [13], as the first, reviewed the performance of PCtn in both postoperative and preoperative detection of MTC. Even if no meta-analysis was performed, the results of PCtn seemed encouraging to use PCtn in clinical practice. Another study [14] focused on PCtn found 15 studies with 485 cases and reported that PCtn seems to be a useful biomarker for both diagnosis and follow-up of MTC when used in conjunction with Ctn, particularly in the small setting of non-secretory MTC. However, the authors underlined that there has not been enough data about the PCtn threshold to be used. The third one [16] aimed at evaluating the performance of PCtn as predictor of MTC in thyroid nodule patients and indicative of response to treatments in previously treated MTC patients. In general, PCtn threshold between 0.06 and 0.50 ng/mL showed sensitivity and specificity of 90% (95% CI: 71–97) and 100% (95% CI: 85–100), respectively. Also, considering PCtn as indicator of relapse (calculated on 4 studies), its sensitivity and specificity were 93% (95% CI: 85–97) and 91% (95% CI: 19–100), respectively.

Table 2 summarizes the findings of these systematic reviews.

**Table 2:** Summary of findings of other markers than calcitonin in diagnosing MTC and/or detecting its relapse

First author	Year	Studies included	MTC included	With/without meta-analysis	Main findings
Meijer [12]	2010	10 about CEA	73 after surgery	With	Both Ctn and CEA doubling time are strong prognostic indicators for MTC recurrence and death with higher performance for CEA.
Trimboli [13]	2016	9 about PCtn	265 after surgery, 114 before surgery	Without	Published data are indicative of a potentially important role of PCtn in the management of MTC patients but it is necessary to establish a diagnostic cutoff.
Karagiannis [14]	2016	15 about PCtn	485 after or before surgery	Without	Ctn continues to be the primary biomarker in MTC. The addition of PCtn may be considered an adjunct to Ctn in the management of patients with MTC.
Trimboli [15]	2018	5 about PCtn	296 after surgery	With	PCtn is reliable to manage MTC patients during their postoperative follow-up.
Giovanella [16]	2021	11 about PCtn	336 after or before surgery	With	PCtn has potential to replace Ctn for the management of MTC patients.
Zarkesh [17]	2022	11 about PCtn, 5 about ProGRP, 3 about Ca 19-9, and 6 about CgA	NA	Without	Ctn is the most sensitive pre- and postoperative marker while CEA can be useful in assessing disease progression. The combination of multiple markers could improve diagnostic sensitivity and specificity.

*MTC* medullary thyroid carcinoma, *Ctn* calcitonin, *CEA* carcinoembryonic antigen, *PCtn* procalcitonin, *Ca 19-9* carbohydrate antigen 19-9, *CgA* chromogranin A, *Pro-GRP* pro-gastrin releasing peptide

### Ultrasound

Three systematic reviews [18–20] aimed at evaluating the US presentation of MTC. The first one [18] reported the performance of US in detecting MTC. This study included 6 original papers reporting the frequency in MTCs of US features generally recognized as suspicious in a thyroid nodule, also in comparison with papillary carcinoma (PTC). The major findings were that suspicious US markers can be useful in diagnosing MTC but with lower performance when compared to PTC. In particular, the frequency of hypoechogenicity, marked hypoechogenicity, microcalcifications, macrocalcifications, absent or irregular halo, solid composition, irregular margins, and taller-than-wide shape in MTCs were 83.4% (95% CI: 46.5–100), 32.7% (95% CI: 21.8–49.1), 35.5% (95% CI: 25.8–49.0), 27.0% (95% CI: 18.3–39.8), 89.9% (95% CI: 31.9–100), 79.2% (95% CI: 51.8–100), 38.0% (95% CI: 21.4–67.4), and 14.4% (95% CI: 8.6–24.2), respectively. Later, Valderrabano et al. [19] reported their institutional series of MTCs and performed an analysis pooling their data with findings from other 9 previously published studies (some included in the study by Woliński [18]). This meta-analysis found that MTC was solid in 92.5% (95% CI: 89.4–95.7), hypoechoic in 96.1% (95% CI: 93.6–98.6), with irregular margins in 38.7% (95% CI: 33.2–44.3), with taller-than-wide shape in 11.1% (95% CI: 7.2–15.1), with microcalcifications in 31.7% (95% CI: 26.1–43.9), and with macrocalcifications in 26% (95% CI: 20.7–31.3). In addition, when compared with PTC, MTC was less likely to have irregular margins, microcalcifications, and taller-than-wide shape, while it had more frequently hypoechogenicity and macrocalcifications. Finally, the meta-analysis by Ferrarazzo et al. [20] included the largest number of studies and aimed to summarize the results of US both considering or not risk stratification systems (RSSs)/TIRADSs, and suspicious US presentation not considering RSSs. There were 1309 MTCs classified according to at least one RSS/TIRADS and 54.8% (95% CI: 48.2–61.5) of them was put in a high‐risk/suspicion category. When considering only ATA system (4 studies and 340 MTCs), MTC was classified at high or intermediate suspicion in 65.1% (95% CI: 50.1–80.1) and 24.9% (95% CI: 12.2–37.6), respectively. There was a subseries of studies not using RSSs/TIRADSs (5 studies and 428 MTCs) and the pooled rate of MTC classified as suspicious was 60.5% (95% CI: 45.1–76). Finally, there were no sufficient data to perform a meta-analysis about the correct indication for FNAC according to RSSs/TIRADSs. Table 3 summarizes the findings of these systematic reviews.

**Table 3:** Summary of findings of US performance in diagnosing MTC

First author	Year	Studies included	MTC included	With/without meta-analysis	Main findings
Woliński [18]	2014	6	169	With	US features commonly considered as suspicious are found in MTC but with less reliability with respect to PTC.
Valderrabano [19]	2016	10	249	With	Suspicious US features are found in MTC differently from PTC.
Ferrarazzo [20]	2022	25	1698	With	According to RSSs/TIRADSs, MTC is classified at high risk/suspicion in just over half of cases.

*US* ultrasound, *MTC* medullary thyroid carcinoma, *PTC* papillary thyroid carcinoma, *TIRADS* thyroid imaging and reporting data system, *ATA* American Thyroid Association, *RSS* risk stratification system

### Fine-needle aspiration

Three systematic reviews were published about FNA in diagnosing MTC [21–23]. Even if the first author was the same, these three studies had different aims and included different studies. The first one [21] aimed at estimating the performance of cytological examination in detecting MTC. There were 641 cases from 15 studies and the pooled rate of FNA samples read as suspicious for of consistent with MTC was 56.4% (95% CI 52.6–60.1). The second systematic review [22] explored the performance of measuring Ctn in washout fluids from biopsy (FNA-Ctn) to detect MTC. This study included 12 studies and concluded, without a meta-analysis, that most MTCs can be correctly detected by FNA-Ctn. The third systematic review [23] was conceived to perform a head-to-head comparison between FNA-cytology and FNA-Ctn to detect MTC. There were 6 articles and 173 MTC lesions undergone biopsy. The low performance of FNA-cytology previously found [21] was confirmed with sensitivity of 54.2% (95% CI: 34.9–73.5), and the performance of FNA-Ctn was found as significantly higher with sensitivity of 98.1% (95% CI: 96.1–100). Table 4 summarizes the findings of these systematic reviews.

**Table 4:** Summary of findings of FNA performance in diagnosing MTC

First author	Year	Studies included	MTC included	With/without meta-analysis	Main findings
Trimboli [21]	2015	15	641	With	MTC is correctly suspected in just over half of cytological samples from biopsy.
Trimboli [22]	2016	12	95	Without	Measuring Ctn in washout fluid from biopsy can be used to detect MTC.
Trimboli [23]	2022	6	173	With	FNA-Ctn has significantly higher performance in detecting MTC than cytology.

*MTC* medullary thyroid carcinoma, *Ctn* calcitonin, *FNA* fine-needle aspiration

### Other imaging procedures (PET, CT, MR)

Six evidence-based articles assessed the role of molecular imaging using PET or hybrid PET/CT with different radiopharmaceuticals in MTC patients, in particular in the restaging setting (suspicious recurrent MTC) [24–29].

Tumors are usually characterized by increased glucose metabolism and this is the rationale for the use of fluorine-18 fluorodeoxyglucose ([^18^F]FDG), a radiolabelled glucose analogue, as PET radiotracer for tumor detection. Two systematic reviews were focused on the role of [^18^F]FDG PET or PET/CT in the detection of recurrent MTC [28, 29]. One study reported a pooled sensitivity of [^18^F]FDG PET and PET/CT of 68% (95% CI: 64–72) and 69% (95% CI: 64–74), respectively [29]. Another meta-analysis demonstrated that the detection rate (DR) of [^18^F]FDG PET or PET/CT in suspicious recurrent MTC on a per patient-based analysis was 59% (95% CI: 54–63). This DR increased in MTC patients with serum Ctn ≥ 1000 ng/L (75%), CEA ≥ 5 ng/ml (69%), Ctn doubling time < 12 months (76%), and CEA doubling time < 24 months (91%) [28].

MTC is characterized by increased uptake of decarboxylation of amine precursors. Then, some studies reported the use of fluorine-18 dihydroxyphenylalanine ([^18^F]FDOPA), a radiolabelled amino acid that is converted to dopamine by aromatic amino acid decarboxylase, in MTC patients. A systematic review and meta-analysis evaluated the performance of [^18^F]FDOPA PET or PET/CT in patients with recurrent MTC and found a DR in per patient- and per lesion-based analysis of 66% (95% CI: 58–74) and 71% (95% CI: 67–75), respectively. This DR increased significantly in MTC patients with serum Ctn ≥1000 ng/L (86%) and Ctn doubling times <24 months (86%) [27].

Since MTC cells, as the other neuroendocrine tumors, may overexpress somatostatin receptors, some studies the use of PET or PET/CT with radiolabelled somatostatin analogues in MTC patients. Here, we found two systematic reviews on this topic. One systematic review reported that the DR of somatostatin receptor PET or PET/CT on a per patient-based analysis was 63.5% (95% CI: 49–77) and increased in patients with higher serum Ctn levels (83% for Ctn >500 ng/L) [26]. This value was lower compared to the diagnostic performance of this method in other neuroendocrine tumors due to the variable expression of somatostatin receptors in MTC. Another systematic review demonstrated that there was no significant difference in number of lesions detected by [^18^F]FDG PET compared with somatostatin receptor PET in recurrent MTC [24].

Lastly, a network meta-analysis comparing five different PET radiopharmaceuticals demonstrated that [^18^F]FDOPA PET/CT clearly showed the best performance for the detection of recurrent MTC in both patient- and lesion-based analyses regardless of serum Ctn or CEA levels and calcitonin doubling time [25].

Table 5 summarizes the findings of these systematic reviews.

**Table 5:** Summary of findings of PET/CT performance in diagnosing MTC and/or detecting its relapse

First author	Year	Studies included	MTC included	With/without meta-analysis	Main findings
Cheng [29]	2012	15	428	With	[^18^F]FDG PET or PET/CT has reasonable sensitivity in detecting recurrent MTC (maximum pooled values of 69%)
Treglia [28]	2012	24	538	With	The DR of [^18^F]FDG PET or PET/CT in recurrent MTC is not optimal but these imaging methods could be helpful in patients with more aggressive disease.
Treglia [27]	2012	8	146	With	[^18^F]FDOPA PET and PET/CT may be useful functional imaging methods in detecting recurrent MTC. The DR of recurrent MTC increases in MTC patients with higher Ctn levels and lower Ctn doubling times.
Treglia [26]	2017	9	152	With	In patients with recurrent MTC, the DR of somatostatin receptor PET or PET/CT is not optimal but it increases in patients with higher serum Ctn values.
Lee [25]	2020	14	306	With	[^18^F]FDOPA clearly shows the best performance for the detection of recurrent MTC among the available PET radiopharmaceuticals.
Pajak [24]	2022	5	113	With	There is no significant difference in number of lesions detected by [^18^F]FDG PET compared with somatostatin receptor PET in recurrent MTC.

*MTC* medullary thyroid carcinoma, *[*^*18*^*F]FDG* fluorine-18 fluorodeoxyglucose, *[18* *F]FDOPA* fluorine-18 dihydroxyphenylalanine, *Ctn* calcitonin, *DR* detection rate

Regarding other imaging procedures, such as CT and MR, no data were found in the systematic reviews included in this study.

## Discussion

The diagnosis of MTC is still challenging and the procedures to detect this rare cancer are still highly debated. The present umbrella review was aimed at summarizing the most important evidence-based information about the performance of Ctn and other circulating markers, US, FNA, and other imaging procedure, in detecting MTC. Both diagnosis of primary MTC and detection of MTC metastases were considered. The summary of findings of the present review is reported in Table 6. The results of this study merit accurate discussion and, from the clinical point of view, their implications in preoperative and postoperative phase should be discusses separately.

**Table 6:** Summary of findings of the present umbrella review

Item	Main finding	Reference supporting the finding
Ctn	Ctn is the most reliable diagnostic marker of MTC. Ctn Ca stimulation test may be useful, when appropriate, but there is no solid evidence about its diagnostic improvement.	[7–9]
	Sometime, MTC presents with normal Ctn (i.e., non-secretory MTC) and these cases may have poorer prognosis.	[10, 11]
Other circulating markers	CEA doubling time is more reliable than that of Ctn in identifying progression of disease and poorer prognosis.	[12, 17]
	PCtn may be useful in adjunct to Ctn to diagnose MTC but there is still no fixed cut-off to be used.	[13–17]
US	The US features commonly considered as suspicious for thyroid malignancy are less reliable in diagnosing MTC.	[18–20]
	MTC is classified at high risk/suspicion according to RSSs/TIRADSs in just over half of cases.	[20]
FNA	FNA cytology can correctly detect MTC in just over half of cases.	[21, 23]
	Measuring Ctn in washout fluid from FNA (FNA-Ctn) has significantly higher performance in detecting MTC than cytology.	[23]
Other imaging procedures	PET/CT (in particular using [^18^F]FDOPA as radiopharmaceutical) is useful for detecting recurrent MTC	[24–29]

*MTC* medullary thyroid carcinoma, *Ctn* calcitonin, *CEA* carcinoembryonic antigen, *PCtn* procalcitonin, *US* ultrasound, *TIRADS* thyroid imaging and reporting data system, *RSS* risk stratification system, *FNA* fine-needle aspiration, *CT* computed tomography, *PET* positron emission tomography, *[*^*18*^*F]FDG* fluorine-18 fluorodeoxyglucose, *[*^*18*^ *F]FDOPA* fluorine-18 dihydroxyphenylalanine

First, Ctn is recognized as the most accurate tool to diagnose MTC and the data found by the present umbrella review fully confirm that. In clinical practice, there is the dilemma about whether testing or not for Ctn all patients with thyroid nodule [30]. The recommendations from international guidelines are quite discrepant. 2006 European Thyroid Association (ETA) guidelines consensus were in favor of routine Ctn measurement [2], 2010 ETA, American Association of Clinical Endocrinologists (AACE) and Associazione Medici Endocrinologi (AME) guidelines recommended for a clinically-oriented use of Ctn [4], and 2015 ATA experts board was neither for neither against its routine measurement [5]. Here we achieved solid evidence about US and FNA cytology having strong implication in the discussion about Ctn measurement. In clinical practice, we generally consider US (and RSS/TIRADS, of course) as highly reliable to identify nodules at risk of malignancy. However, we must take into account that all the information we have about US (and RSS/TIRADS, as a consequence) has been substantially achieved considering PTC [31]. In fact, the largest part of studies evaluating the performance of RSSs/TIRADSs in detecting thyroid malignancy has adopted cytology as the gold standard. This represents a strong bias because cytology is optimal in diagnosing PTC, while MTC can be detected on cytological specimens in just above a half of cases and follicular carcinoma cannot be identified by FNA. To summarize, we strongly need new strategies to measure Ctn appropriately and, when indicated, select nodules for FNA (with the essential Ctn measurement in fluids from FNA). Regarding the other circulating markers there is some evidence in favor of PCtn, even if at present we have no fixed cut-off to use in clinical practice. However, testing for PCtn in adjunct to Ctn seems to be reasonable in case of doubt.

Second, Ctn is reliable as postoperative marker for detecting MTC relapse. Among the other circulating markers, PCtn shows performance not inferior to Ctn and it can be used in combination to Ctn. Currently, we have no solid proof in favor of using PCtn as a separate marker instead of Ctn since it is precursor of Ctn and its values are strongly correlated with Ctn levels [32]. CEA doubling time is more accurate than Ctn doubling time in discriminating patients at high risk of poor prognosis and TC-specific mortality. From the clinical point of view the measurement of Ctn, PCtn, and CEA is essential during postoperative follow-up of all MTC patients. Regarding the imaging procedures, the present umbrella review achieves solid evidence that [^18^F]FDG PET combined or not with CT, even if it is associated with suboptimal DR of metastases from MTC, has optimal performance in identifying patients with more aggressive disease. In addition, the present umbrella review demonstrates that, among the different imaging procedures, [^18^F]FDOPA has the highest performance in detecting recurrent MTC. No evidence-based data were found about CT and MR. This is of great interest for clinical practice because CT has to be considered to stage and restage high-risk MTC with extensive neck disease and high Ctn levels. In fact, MTC ATA guidelines [32] recommend CT in cases with high probability of distant metastases when Ctn levels exceed 500 pg/mL at initial diagnosis or 150 pg/mL during postoperative follow-up. Furthermore, ATA guidelines [33] suggest using MR in patients suspected for metastases in liver and axial skeleton. In this context, it has to be cited that three recent narrative reviews [34–36] reported that (1) CT can detect mediastinal lymph node metastases; (2) CT is a sensitive procedure to identify pulmonary metastases that may appear small and diffuse to both lungs; (3) multiparametric MR is highly effective in disclosing small liver metastases, and in this field, it should be proposed as the first-line imaging procedure. By considering the lack of evidence-based data focused on these morphological imaging procedures and the increasing amount of data regarding the opportunity to use molecular imaging procedures in patients affected by MTC, it seems reasonable to propose [^18^F]FDOPA PET/CT as the most appropriate imaging tool to properly restage patients at high risk to develop neck lymph-nodes or distant metastases. Indeed, when [^18^F]FDOPA PET/CT is clinically available, its use should be always considered in this kind of patients [37].

This umbrella review has some limitations mainly related to the included systematic reviews. First, in some eligible systematic reviews, a limited number of original studies and MTC patients were included influencing the statistical power and the strength of the results of the related meta-analysis. Second, heterogeneity was reported in some included systematic reviews/meta-analyses mainly due to differences among the included studies in terms of quality, study design, characteristics of patients included, methods, and reference standard. Even if biases cannot be excluded, awareness of the results described in this umbrella review may affect MTC patient care by providing supportive evidence for more effective use of several diagnostic tools.

In conclusion, the present data can allow to report that (1) Ctn remains the most reliable tool to diagnose MTC, (2) CEA doubling time is essential during the postoperative follow-up to identify patients at high risk of death, (3) US features, and TIRADSs, are not sufficiently reliable to diagnose MTC, (4) cytological examination has poor sensitivity in detecting MTC and FNA-Ctn is essential to avoid false negative biopsy, and (5) PET/CT (in particular using [^18^F]FDOPA as radiopharmaceutical) is useful for detecting recurrent MTC.

## Supplementary information

Supplementary Information

## Data Availability

The datasets generated during and/or analyzed during the current study are not publicly available but are available from the corresponding author on reasonable request.

## References

[1] Tuttle RM, Ball DW, Byrd D (2010). Medullary carcinoma. J. Natl Compr. Cancer Netw..

[2] Pacini F, Schlumberger M, Dralle H, Elisei R, Smit JW, Wiersinga W (2006). European Thyroid Cancer Taskforce. European consensus for the management of patients with differentiated thyroid carcinoma of the follicular epithelium. Eur. J. Endocrinol..

[3] Kloos RT, Eng C, Evans DB, Francis GL, Gagel RF, Gharib H, Moley JF, Pacini F, Ringel MD, Schlumberger M (2009). Medullary thyroid cancer: management guidelines of the American Thyroid Association. Thyroid.

[4] Gharib H, Papini E, Paschke R (2010). American Association of Clinical Endocrinologists, Associazione Medici Endocrinologi, and European Thyroid Association medical guidelines for clinical practice for the diagnosis and management of thyroid nodules: executive summary of recommendations. J. Endocrinol. Invest.

[5] Haugen BR, Alexander EK, Bible KC, Doherty GM, Mandel SJ, Nikiforov YE, Pacini F, Randolph GW, Sawka AM, Schlumberger M, Schuff KG, Sherman SI, Sosa JA, Steward DL, Tuttle RM, Wartofsky L (2016). 2015 American Thyroid Association management guidelines for adult patients with thyroid nodules and differentiated thyroid cancer: the American Thyroid Association guidelines task force on thyroid nodules and differentiated thyroid cancer. Thyroid.

[6] Trimboli P, Crescenzi A, Saggiorato E, Treglia G, Giovanella L (2017). Novel acquisitions in the diagnosis of medullary thyroid carcinoma. Minerva Endocrinol..

[7] Verbeek HH, de Groot JWB, Sluiter WJ, Muller Kobold AC, van den Heuvel ER, Plukker JT, Links TP (2020). Calcitonin testing for detection of medullary thyroid cancer in people with thyroid nodules. Cochrane Database Syst. Rev..

[8] Vardarli I, Weber M, Weidemann F, Führer D, Herrmann K, Görges R (2021). Diagnostic accuracy of routine calcitonin measurement for the detection of medullary thyroid carcinoma in the management of patients with nodular thyroid disease: a meta-analysis. Endocr. Connect.

[9] Băetu M, Olariu CA, Moldoveanu G, Corneci C, Badiu C (2021). Calcitonin stimulation tests: rationale, technical issues and side effects: a review. Horm. Metab. Res..

[10] Trimboli P, Giovanella L (2015). Serum calcitonin negative medullary thyroid carcinoma: a systematic review of the literature. Clin. Chem. Lab Med.

[11] Gambardella C, Offi C, Patrone R, Clarizia G, Mauriello C, Tartaglia E, Di Capua F, Di Martino S, Romano RM, Fiore L, Conzo A, Conzo G, Docimo G (2019). Calcitonin negative Medullary Thyroid Carcinoma: a challenging diagnosis or a medical dilemma?. BMC Endocr. Disord..

[12] Meijer JA, le Cessie S, van den Hout WB, Kievit J, Schoones JW, Romijn JA, Smit JW (2010). Calcitonin and carcinoembryonic antigen doubling times as prognostic factors in medullary thyroid carcinoma: a structured meta-analysis. Clin. Endocrinol. (Oxf.).

[13] Trimboli P, Seregni E, Treglia G, Alevizaki M, Giovanella L (2015). Procalcitonin for detecting medullary thyroid carcinoma: a systematic review. Endocr. Relat. Cancer.

[14] Karagiannis AK, Girio-Fragkoulakis C, Nakouti T (2016). Procalcitonin: a new biomarker for medullary thyroid cancer? A systematic review. Anticancer Res..

[15] Trimboli P, Giovanella L (2018). Procalcitonin as marker of recurrent medullary thyroid carcinoma: a systematic review and meta-analysis. Endocrinol. Metab. (Seoul.)..

[16] Giovanella L, Garo ML, Ceriani L, Paone G, Campenni’ A, D’Aurizio F (2021). Procalcitonin as an Alternative Tumor Marker of Medullary Thyroid Carcinoma. J. Clin. Endocrinol. Metab..

[17] Zarkesh M, Arab N, Tavangar SM, Nozhat Z, Fanaei SM, Hedayati M (2022). Utilizing the circulating tumor markers in diagnosis and management of medullary thyroid cancer. Pathol. Res Pr..

[18] Woliński K, Rewaj-Łosyk M, Ruchała M (2014). Sonographic features of medullary thyroid carcinomas–a systematic review and meta-analysis. Endokrynol. Pol..

[19] Valderrabano P, Klippenstein DL, Tourtelot JB, Ma Z, Thompson ZJ, Lilienfeld HS, McIver B (2016). New American Thyroid Association sonographic patterns for thyroid nodules perform well in medullary thyroid carcinoma: institutional experience, systematic review, and meta-analysis. Thyroid.

[20] Ferrarazzo G, Camponovo C, Deandrea M, Piccardo A, Scappaticcio L, Trimboli P (2022). Suboptimal accuracy of ultrasound and ultrasound-based risk stratification systems in detecting medullary thyroid carcinoma should not be overlooked. Findings from a systematic review with meta-analysis. Clin. Endocrinol. (Oxf.).

[21] Trimboli P, Treglia G, Guidobaldi L, Romanelli F, Nigri G, Valabrega S, Sadeghi R, Crescenzi A, Faquin WC, Bongiovanni M, Giovanella L (2015). Detection rate of FNA cytology in medullary thyroid carcinoma: a meta-analysis. Clin. Endocrinol. (Oxf.).

[22] Trimboli P, Guidobaldi L, Bongiovanni M, Crescenzi A, Alevizaki M, Giovanella L (2016). Use of fine-needle aspirate calcitonin to detect medullary thyroid carcinoma: A systematic review. Diagn. Cytopathol..

[23] Trimboli P, Giannelli J, Marques B, Piccardo A, Crescenzi A, Deandrea M (2022). Head-to-head comparison of FNA cytology vs. calcitonin measurement in FNA washout fluids (FNA-CT) to diagnose medullary thyroid carcinoma. A systematic review and meta-analysis. Endocrine.

[24] Pajak C, Cadili L, Nabata K, Wiseman SM (2022). 68Ga-DOTATATE-PET shows promise for diagnosis of recurrent or persistent medullary thyroid cancer: A systematic review. Am. J. Surg..

[25] Lee SW, Shim SR, Jeong SY, Kim SJ (2020). Comparison of 5 different PET radiopharmaceuticals for the detection of recurrent medullary thyroid carcinoma: a network meta-analysis. Clin. Nucl. Med.

[26] Treglia G, Tamburello A, Giovanella L (2017). Detection rate of somatostatin receptor PET in patients with recurrent medullary thyroid carcinoma: a systematic review and a meta-analysis. Hormones (Athens).

[27] Treglia G, Cocciolillo F, Di Nardo F, Poscia A, de Waure C, Giordano A, Rufini V (2012). Detection rate of recurrent medullary thyroid carcinoma using fluorine-18 dihydroxyphenylalanine positron emission tomography: a meta-analysis. Acad. Radio..

[28] Treglia G, Villani MF, Giordano A, Rufini V (2012). Detection rate of recurrent medullary thyroid carcinoma using fluorine-18 fluorodeoxyglucose positron emission tomography: a meta-analysis. Endocrine.

[29] Cheng X, Bao L, Xu Z, Li D, Wang J, Li Y (2012). ^18^F-FDG-PET and ^18^F-FDG-PET/CT in the detection of recurrent or metastatic medullary thyroid carcinoma: a systematic review and meta-analysis. J. Med Imaging Radiat. Oncol..

[30] Trimboli P, Camponovo C, Ruinelli L (2022). The dilemma of routine testing for calcitonin thyroid nodule’s patients to detect or exclude medullary carcinoma: one single negative test should be valuable as rule-out strategy to avoid further calcitonin measurements over time. Endocrine.

[31] Trimboli P, Castellana M, Piccardo A, Romanelli F, Grani G, Giovanella L, Durante C (2021). The ultrasound risk stratification systems for thyroid nodule have been evaluated against papillary carcinoma. A meta-analysis. Rev. Endocr. Metab. Disord..

[32] Woliński K, Kaznowski J, Klimowicz A, Maciejewski A, Łapińska-Cwojdzińska D, Gurgul E, Car AD, Fichna M, Gut P, Gryczyńska M, Ruchała M (2017). Diagnostic value of selected biochemical markers in the detection of recurrence of medullary thyroid cancer - comparison of calcitonin, procalcitonin, chromogranin A, and carcinoembryonic antigen. Endokrynol. Pol..

[33] Wells SA, Asa SL, Dralle H, Elisei R, Evans DB, Gagel RF, Lee N, Machens A, Moley JF, Pacini F, Raue F, Frank-Raue K, Robinson B, Rosenthal MS, Santoro M, Schlumberger M, Shah M, Waguespack SG, American Thyroid Association Guidelines Task Force on Medullary Thyroid Carcinoma (2015). Revised American Thyroid Association guidelines for the management of medullary thyroid carcinoma. Thyroid.

[34] Klain M, Hadoux J, Nappi C, Finessi M, Ambrosio R, Schlumberger M, Cuocolo A, Deandreis D, Salvatore D (2022). Imaging medullary thyroid cancer patients with detectable serum markers: state of the art and future perspectives. Endocrine.

[35] Leimbach RD, Hoang TD, Shakir MKM (2021). Diagnostic challenges of medullary thyroid carcinoma. Oncology.

[36] Kushchayev SV, Kushchayeva YS, Tella SH, Glushko T, Pacak K, Teytelboym OM (2019). Medullary thyroid carcinoma: an update on imaging. J. Thyroid Res.

[37] Giovanella L, Treglia G, Iakovou I, Mihailovic J, Verburg FA, Luster M (2020). EANM practice guideline for PET/CT imaging in medullary thyroid carcinoma. Eur. J. Nucl. Med Mol. Imaging.

