# Supplementary material for: Diagnostic tests for medullary thyroid carcinoma: an umbrella review
Source: Endocrine. 2023 Mar 6;81(2):183–93. doi: 10.1007/s12020-023-03326-6 (PMC10293444; doi:10.1007/s12020-023-03326-6)
Supplement: Supplementary file 1 — Supplementary Information [file 12020_2023_3326_MOESM1_ESM.docx]

# Quality assessment (AMSTAR 2)* of the included systematic reviews

Items judged of critical importance: comprehensiveness of the literature search; included studies and reasons for excluding studies described in adequate detail; adequacy of risk of bias assessment of primary studies; appropriateness of meta-analytical methods (only when a meta-analysis is performed); consideration of risk of bias when interpreting the results of the review. According to the responses to critical items, a final overall judgment of the whole review can be:

High quality: No or one non-critical weakness: the systematic review provides an accurate and comprehensive summary of the results of the available studies that address the question of interest

Moderate quality: More than one non-critical weakness but no critical flaws. It may provide an accurate summary of the results of the available studies that were included in the review

Low quality: One critical flaw with or without non-critical weaknesses: the review may not provide an accurate and comprehensive summary of the available studies that address the question of interest

Critically low quality: More than one critical flaw with or without non-critical weaknesses: the review should not be relied on to provide an accurate and comprehensive summary of the available studies

*****Shea, B.J., et al., AMSTAR 2: a critical appraisal tool for systematic reviews that include randomised or non-randomised studies of healthcare interventions, or both. BMJ, 2017. 358: p. j4008

# Systematic reviews on laboratory biomarkers in medullary thyroid cancer

**Quality assessment of the included systematic reviews**

| n | AMSTAR-2 criteria | **Baetu et al. 2021 [9]** | **Gambardella et al. 2019 [11]** | **Giovanella et al. 2021 [16]** | **Karagiannis et al. 2016 [14]** | **Meijer et al. 2010 [12]** | **Trimboli et al. 2015 [10]** | **Trimboli et al. 2015 [13]** |
| --- | --- | --- | --- | --- | --- | --- | --- | --- |
| 1 | Research questions and inclusion criteria include components of PICO | yes | yes | yes | yes | yes | yes | yes |
| 2 | Review methods established prior to the conduct of the review (protocol) and deviations justified | partial yes | partial yes | yes | partial yes | partial yes | partial yes | partial yes |
| 3 | Selection of study design explained | yes | yes | yes | yes | yes | yes | yes |
| 4 | Comprehensive literature search strategy | no (PubMed only) | no (PubMed only) | yes | yes | yes | yes | yes |
| 5 | Study selection in duplicate | not reported | yes | yes | not reported | not reported | yes | yes |
| 6 | Data extraction in duplicate | not reported | not reported | yes | not reported | not reported | yes | yes |
| 7 | List of excluded studies and justification of the exclusions | no | yes | yes | yes | no | no | no |
| 8 | Included studies described in adequate detail | yes | yes | yes | yes | yes | yes | yes |
| 9 | Technique for assessing the risk of bias satisfactory | no | yes | yes | no | no | no | no |
| 10 | Sources of funding for the primary studies reported | no | no | no | no | no | no | no |
| 11 | Appropriate methods for meta-analysis | no meta-analysis conducted | no meta-analysis conducted | yes | no meta-analysis conducted | yes | no meta-analysis conducted | no meta-analysis conducted |
| 12 | Potential impact of risk of bias results on meta-analysis assessed | no meta-analysis conducted | no meta-analysis conducted | yes | no meta-analysis conducted | no | no meta-analysis conducted | no meta-analysis conducted |
| 13 | Risk of bias results accounted for in discussion/conclusion | no | no | yes | no | yes | no | no |
| 14 | Satisfactory discussion and explanation of observed heterogeneity, if any | no | no | yes | no | yes | no | yes |
| 15 | Adequate investigation of publication bias | no meta-analysis conducted | no meta-analysis conducted | yes | no meta-analysis conducted | not reported | no meta-analysis conducted | yes |
| 16 | Conflict of interest of review authors and funding received for conducting the review reported | yes | yes | yes | not reported | yes | yes | yes |
|  | Overall methodological quality | **low** | **low** | **high** | **low** | **low** | **low** | **low** |

| n | AMSTAR-2 criteria | **Trimboli et al. 2018 [15]** | **Vardarli et al. 2021 [8]** | **Verbeek et al. 2020 [7]** | **Zarkesh et al. 2022 [17]** |
| --- | --- | --- | --- | --- | --- |
| 1 | Research questions and inclusion criteria include components of PICO | yes | yes | yes | yes |
| 2 | Review methods established prior to the conduct of the review (protocol) and deviations justified | partial yes | yes | yes | yes partial |
| 3 | Selection of study design explained | yes | yes | yes | yes |
| 4 | Comprehensive literature search strategy | yes | yes | yes | yes |
| 5 | Study selection in duplicate | yes | yes | yes | not reported |
| 6 | Data extraction in duplicate | yes | yes | yes | not reported |
| 7 | List of excluded studies and justification of the exclusions | yes | yes | yes | yes |
| 8 | Included studies described in adequate detail | yes | yes | yes | yes |
| 9 | Technique for assessing the risk of bias satisfactory | yes | yes | yes | no |
| 10 | Sources of funding for the primary studies reported | no | no | yes | no |
| 11 | Appropriate methods for meta-analysis | yes | yes | yes | no meta-analysis conducted |
| 12 | Potential impact of risk of bias results on meta-analysis assessed | yes | yes | yes | no meta-analysis conducted |
| 13 | Risk of bias results accounted for in discussion/conclusion | yes | yes | yes | no |
| 14 | Satisfactory discussion and explanation of observed heterogeneity, if any | yes | yes | yes | no |
| 15 | Adequate investigation of publication bias | yes | yes | not reported | no meta-analysis conducted |
| 16 | Conflict of interest of review authors and funding received for conducting the review reported | yes | yes | yes | yes |
|  | Overall methodological quality | **moderate** | **high** | **high** | **low** |

# Systematic reviews on ultrasound in medullary thyroid cancer

**Quality assessment of the included systematic reviews**

| n | AMSTAR-2 criteria | **Ferrarazzo et al. 2022 [20]** | **Valderrabano et al. 2016 [19]** | **Wolinski et al. 2014 [18]** |
| --- | --- | --- | --- | --- |
| 1 | Research questions and inclusion criteria include components of PICO | yes | yes | yes |
| 2 | Review methods established prior to the conduct of the review (protocol) and deviations justified | partial yes | partial yes | partial yes |
| 3 | Selection of study design explained | yes | yes | yes |
| 4 | Comprehensive literature search strategy | yes | no (one database) | yes |
| 5 | Study selection in duplicate | yes | no | yes |
| 6 | Data extraction in duplicate | yes | no | yes |
| 7 | List of excluded studies and justification of the exclusions | yes | yes | yes |
| 8 | Included studies described in adequate detail | yes | yes | yes |
| 9 | Technique for assessing the risk of bias satisfactory | yes | no | no |
| 10 | Sources of funding for the primary studies reported | no | no | no |
| 11 | Appropriate methods for meta-analysis | yes | yes | yes |
| 12 | Potential impact of risk of bias results on meta-analysis assessed | yes | no | yes |
| 13 | Risk of bias results accounted for in discussion/conclusion | yes | no | no |
| 14 | Satisfactory discussion and explanation of observed heterogeneity, if any | yes | no | yes |
| 15 | Adequate investigation of publication bias | no | no | no |
| 16 | Conflict of interest of review authors and funding received for conducting the review reported | yes | yes | yes |
|  | Overall methodological quality | **moderate** | **low** | **low** |

# Systematic reviews on fine needle aspiration in medullary thyroid cancer

**Quality assessment of the included systematic reviews**

| n | AMSTAR-2 criteria | **Trimboli et al. 2015 [21]** | **Trimboli et al. 2016 [22]** | **Trimboli et al. 2022 [23]** |
| --- | --- | --- | --- | --- |
| 1 | Research questions and inclusion criteria include components of PICO | yes | yes | yes |
| 2 | Review methods established prior to the conduct of the review (protocol) and deviations justified | partial yes | partial yes | partial yes |
| 3 | Selection of study design explained | yes | yes | yes |
| 4 | Comprehensive literature search strategy | yes | yes | yes |
| 5 | Study selection in duplicate | yes | yes | yes |
| 6 | Data extraction in duplicate | yes | yes | yes |
| 7 | List of excluded studies and justification of the exclusions | yes | no | yes |
| 8 | Included studies described in adequate detail | yes | yes | yes |
| 9 | Technique for assessing the risk of bias satisfactory | no | no | yes |
| 10 | Sources of funding for the primary studies reported | no | no | no |
| 11 | Appropriate methods for meta-analysis | yes | no meta-analysis conducted | yes |
| 12 | Potential impact of risk of bias results on meta-analysis assessed | yes | no meta-analysis conducted | yes |
| 13 | Risk of bias results accounted for in discussion/conclusion | yes | no | yes |
| 14 | Satisfactory discussion and explanation of observed heterogeneity, if any | yes | no | yes |
| 15 | Adequate investigation of publication bias | yes | no meta-analysis conducted | not reported |
| 16 | Conflict of interest of review authors and funding received for conducting the review reported | yes | yes | yes |
|  | Overall methodological quality | **moderate** | **low** | **high** |

# Systematic reviews on positron emission tomography in medullary thyroid cancer

**Quality assessment of the included systematic reviews**

| n | AMSTAR-2 criteria | **Cheng et al. 2012 [29]** | **Lee et al. 2020 [25]** | **Pajak et al. 2022 [24]** | **Treglia et al. 2012 [28]** | **Treglia et al. 2012 [27]** | **Treglia et al. 2017 [26]** |
| --- | --- | --- | --- | --- | --- | --- | --- |
| 1 | Research questions and inclusion criteria include components of PICO | yes | yes | yes | yes | yes | yes |
| 2 | Review methods established prior to the conduct of the review (protocol) and deviations justified | partial yes | partial yes | partal yes | partial yes | partial yes | partial yes |
| 3 | Selection of study design explained | yes | yes | yes | yes | yes | yes |
| 4 | Comprehensive literature search strategy | yes | yes | yes | yes | yes | yes |
| 5 | Study selection in duplicate | yes | yes | yes | yes | yes | yes |
| 6 | Data extraction in duplicate | yes | yes | yes | yes | yes | yes |
| 7 | List of excluded studies and justification of the exclusions | no | yes | yes | yes | yes | yes |
| 8 | Included studies described in adequate detail | yes | yes | yes | yes | yes | yes |
| 9 | Technique for assessing the risk of bias satisfactory | no | yes | no | yes | yes | yes |
| 10 | Sources of funding for the primary studies reported | no | no | no | no | no | no |
| 11 | Appropriate methods for meta-analysis | yes | yes | yes | yes | yes | yes |
| 12 | Potential impact of risk of bias results on meta-analysis assessed | yes | yes | yes | yes | yes | yes |
| 13 | Risk of bias results accounted for in discussion/conclusion | yes | yes | no | yes | yes | yes |
| 14 | Satisfactory discussion and explanation of observed heterogeneity, if any | yes | yes | yes | yes | yes | yes |
| 15 | Adequate investigation of publication bias | not reported | yes | not reported | not reported | not reported | not reported |
| 16 | Conflict of interest of review authors and funding received for conducting the review reported | yes | yes | yes | yes | yes | yes |
|  | Overall methodological quality | **low** | **high** | **low** | **moderate** | **moderate** | **moderate** |
